# NF-kappaB Regulates Redox Status in Breast Cancer Subtypes

**DOI:** 10.3390/genes9070320

**Published:** 2018-06-26

**Authors:** Bruno R. B. Pires, Renata Binato, Gerson M. Ferreira, Rubens Cecchini, Carolina Panis, Eliana Abdelhay

**Affiliations:** 1 Laboratório de Célula-Tronco, Instituto Nacional de Câncer, Rio de Janeiro-RJ 20230-130, Brazil; renata.binato@inca.gov.br (R.B.); gerson.ferreira@inca.gov.br (G.M.F.); carolinapanis@hotmail.com (C.P.); eabdelhay@inca.gov.br (E.A.); 2 Instituto Nacional de Ciência e Tecnologia para o Controle do Câncer, Rio de Janeiro-RJ 20231-050, Brazil; 3 Laboratório de Fisiopatologia e Radicais Livres, Universidade Estadual de Londrina, Londrina-PR 86057-970, Brazil; cecchini@uel.br; 4 Laboratório de Mediadores Inflamatórios, Universidade Estadual do Oeste do Paraná, Francisco Beltrão-PR 85605-010, Brazil

**Keywords:** breast cancer, subtypes, NFkappaB, redox, oxidative stress, microarray, gene expression

## Abstract

Oxidative stress (OS) is an indispensable condition to ensure genomic instability in cancer cells. In breast cancer (BC), redox alterations have been widely characterized, but since this process results from a chain of inflammatory events, the causal molecular triggers remain to be identified. In this context, we used a microarray approach to investigate the role of the main pro-oxidant transcription factor, nuclear factor-kappa B (NF-κB), in gene profiles of BC subtypes. Our results showed that NF-κB knockdown in distinct BC subtypes led to differential expression of relevant factors involved in glutathione metabolism, prostaglandins, cytochrome P450 and cyclooxygenase, suggesting a relationship between the redox balance and NF-κB in such cells. In addition, we performed biochemical analyses to validate the microarray dataset focusing on OS and correlated these parameters with normal expression or NF-κB inhibition. Our data showed a distinct oxidative status pattern for each of the three studied BC subtype models, consistent with the intrinsic characteristics of each BC subtype. Thus, our findings suggest that NF-κB may represent an additional mechanism related to OS maintenance in BC, operating in various forms to mediate other important predominant signaling components of each BC subtype.

## 1. Introduction

Breast cancer (BC) is a heterogeneous disease composed of multiple subtypes with distinct progressions, outcomes, and molecular features. This neoplasia is the leading cause of cancer-associated death among women worldwide. In the U.S., it is expected that approximately 270,000 new cases of female BC and more than 40,000 deaths will occur in 2018 [1]. Based on gene expression profiles, human BC may be classified in three major intrinsic groups: Luminal, HER2-enriched and triple-negative (TNBC) [2]. The Luminal subtype is characterized by the expression of estrogen and/or progesterone receptors (ER and PR, respectively); HER2-enriched overexpresses HER2/neu (human epidermal growth factor receptor 2); and TNBC shows negative expression for ER, PR and HER2 [2,3]. Recent advances have led to an understanding of the biological events in BC at the molecular level, but many issues must still be addressed. Molecular components from inflammatory networks have risen as major players in BC pathogenesis, especially nuclear factor (NF)-κB-driven signaling [4].

The NF-κB family consists of five conserved proteins, RelA (p65), RelB, c-Rel, p50, and p52. All of them share the conserved Rel homology domain (RHD) responsible for DNA binding, dimerization, and association with the repressor protein IκB. The NF-κB pathway comprises the canonical or noncanonical pathway. The classical pathway is activated by proinflammatory cytokine receptors, such as tumor necrosis factor (TNF)-α, interleukin (IL)-1β, the Toll-like receptor (TLR) family, T cell receptor (TCR), and B cell receptor (BCR), ultimately leading to ubiquitin-dependent degradation of the repressor IκB through phosphorylation of the IKK complex (IKKα, IKKβ and IKKγ). The repressor protein IκB degradation releases the p65/p50 dimer to translocate into the nucleus and activate the transcription of target genes [5,6]. Noncanonical signaling occurs downstream of cluster of differentiation 40 ligand (CD40L) and B cell-activating factor receptor (BAFF-R) pathways that lead to NF-κB-inducing kinase (NIK) activation, resulting in phosphorylation of the homodimer IKKα and transfer of the phosphate group to the p100 subunit to be processed into p52. As a result, nuclear translocation of p52/RelB dimer occurs [5,7].

Since cancer-related inflammation (CRI) was recognized as a hallmark of cancer, NF-κB assumed new importance in cancer studies [8,9]. NF-κB is a regulator of innate immunity and is responsible for inducing the expression of cyclooxygenase (COX)-2 and nitric oxide synthase (NOS), inflammatory cytokines such as IL-1, IL-6, IL-8, and TNF-α, and chemokines such as CCL2 and CXCL8 [4,5]. Regarding BC, constitutive activation of NF-κB contributes to several downstream pathways that regulate cellular proliferation, angiogenesis, evasion of apoptosis, induction of the cancer stem cell phenotype, oxidative stress (OS) and invasiveness [10,11,12,13,14,15,16,17]. Hence, the NF-κB pathway is a promising target for cancer therapy.

Despite wide knowledge about NF-κB in cancer, few studies have focused on understanding its role in the distinct molecular subtypes of BC. Here, we sought to examine the role of NF-κB in BC cells representing the main phenotypes of the disease (Luminal, HER2-enriched and TNBC). Therefore, we performed a series of microarray analyses after NF-κB silencing, aiming to unravel the main downstream pathways regulated by this transcription factor in each BC subtype. Our findings demonstrate that NF-κB has an important role in the redox balance in the studied BC subtype models, suggesting that NF-κB may represent an additional mechanism related to OS maintenance in BC, operating in various forms to mediate other important predominant signaling components of each BC subtype.

## 2. Materials and Methods

### 2.1. Cell Culture and Transfection

The human breast cancer cell lines MDA-MB-231 (ATCC HTB-26, TNBC), HCC-1954 (ATCC CRL-2338, HER2) and MCF-7 (ATCC HTB-22, Luminal) were cultured in RPMI 1640 (Sigma-Aldrich, St. Louis, MO, USA) supplemented with 10% fetal bovine serum (FBS), 2 mM glutamine, 100 units/mL penicillin, and 100 µg/mL streptomycin. The cells were cultured in a humidified 5% CO_2_ atmosphere at 37 °C.

Specific short interference RNA (siRNA) (sc-29410, Santa Cruz Biotechnology, Dallas, TX, USA) was used to knock down the gene expression of NF-κB/p65. An oligonucleotide that did not match any human coding cDNA was used as a negative control (Scramble, sc-37007, Santa Cruz). Transfections were performed using Lipofectamine LTX with Plus Reagent as per the manufacturer’s instructions (Thermo Fisher, Waltham, MA, USA). As previously described [18], 4 × 105 cells were seeded in a 6-well plate and were then transfected with 50 nM of siNF-κB or Scramble for 72 h. The transfected cells were subsequently used for further experiments.

### 2.2. Chemicals

Dehydroxymethylepoxyquinomicin (DHMEQ) was used to inhibit NF-κB/p65. As previously described [18], 10 µg/mL of DHMEQ was used in all experiments. All chemicals used in the oxidative stress analysis were purchased from Sigma-Aldrich.

### 2.3. Expression Chip Array Data Analysis

Total RNA from NF-κB-silenced and non-silenced cells was obtained using the RNeasy Mini kit (Qiagen, Hilden, Germany) following the manufacturer’s instructions. Next, 100 ng of each RNA was used to synthesize and biotinylate cRNA according to the GeneChip whole transcription (WT) sense target-labeling assay (Thermo Fisher). The biotinylated cRNA was hybridized to the GeneChip human gene 1.0 ST array (Thermo Fisher), washed and stained according to the manufacturer’s protocols. The GeneChip arrays were scanned using GeneChip^®^ Scanner 3000. Affymetrix Expression Console™ software v.1.0 (Thermo Fisher) was used to create summarized expression values (CHP-files). Data were analyzed using the Affymetrix Transcriptome Analysis Console (Thermo Fisher), whereby differentially expressed genes with ≥1.5-fold-change were used as criteria to define up-regulation or down-regulation compared with the corresponding Scramble. 

In silico analysis of gene ontology, biological processes and signaling pathways, that were altered as a consequence of NF-κB knockdown, was performed using KEGG [19] and PANTHER software [20]. A pathway enrichment analysis was performed using MetaCore software v.6.35 (Thomson Reuters, Toronto, Canada) A Venn diagram was generated using the online software of Bioinformatics & Evolutionary Genomics [21].

### 2.4. Real-Time Reverse Transcription Polymerase Chain Reaction (RT-qPCR)

Two micrograms of RNA were subjected to the DNAse Amplification Grade I Kit (Thermo Fisher) for removal of DNA contamination and reverse-transcribed into cDNA using the Superscript-III kit (Thermo Fisher) per the manufacturer’s protocol. Real-Time reverse transcription PCR (RT-qPCR) was performed with SYBR Green Master Mix (Thermo Fisher) in a Rotor-Gene Q (Qiagen), and the conditions were as follows: 95 °C for 10 min, followed by 45 cycles of 30 s at 95 °C, 30 s at 60 °C and 30 s at 72 °C. The primers used are described in Table 1. The mean of the housekeeping genes ACTB and GAPDH was used as the reference expression for the mRNA levels. Each sample was examined in triplicate. The fold expression was calculated according to the ΔΔCt method [22].

### 2.5. Biochemical Analysis

Based on the microarray results, we chose to investigate oxidative-stress related genes for the validation step. The oxidative status of the total content of cells and supernatant (5 × 105 cells/mL) was determined by the quantification of total thiol content and estimation of nitric oxide levels by nitrite (NO) and lipid peroxidation profiling. The thiol content was measured by the addition of 320 µL of Tris-EDTA buffer (0.25 M/0.02 M, pH 8.2) and 40 µL of DTNB (5,5′-dithiobis(2-nitrobenzoic acid)—0.01 M) to 50 µL of sample containing cells and supernatant. The resulting yellow compound was measured at 412 nm, and the results are represented as µM of thiol [23]. For NO levels, aliquots of 60 µL of sample containing cells and supernatant were deproteinized in 50 µL of ZnSO_4_ (75 mM), centrifuged at 10,000 rpm for 2 min and then mixed with 70 µL of NaOH (55 mM). After centrifugation at 10,000 rpm for 5 min, 150 µL of supernatant was added to 50 µL of glycine buffer (45 g/L, pH 9.7) and incubated for 10 min with cadmium granules (Fluka, Sigma-Aldrich, St. Louis, MO, USA) previously activated by CuSO_4_ (5 mM, 5 min) to convert nitrate to nitrite. Next, of 50-µL aliquots were mixed with Griess reagent for 10 min, and the absorbance was measured at 550 nm [24]. The results are expressed as µM of nitrite. Lipid peroxidation profiling was determined using 500 µL of sample containing cells and supernatant, supplemented with 500 µL of phosphate buffer (K_2_HPO_4_ 30 mM in KCl 1.15%, pH 7.4, 37 °C) and 20 µL of tert-butyl hydroperoxide (3 mM) [25]. Data were quantified using a Glomax luminometer (Promega, Madison, WI, USA), and the results were analyzed with OriginLab 7.5 (OriginLab) software and expressed as relative light units (RLUs) in 20 min.

### 2.6. Statistical Analysis

All data are expressed as the mean ± standard deviation (SD) of at least three independent experiments and were analyzed by a two-tailed Student’s *t*-test or ANOVA with GraphPad Prism v.5 (GraphPad Inc., San Diego, CA, USA). *p*-values < 0.05 were considered statistically significant.

## 3. Results

### 3.1. NF-κB/p65 Inhibition Altered the Gene Expression Profile of Redox Signaling

We have recently described the importance of NF-κB/p65 in the regulation of epithelial-to-mesenchymal transition (EMT) in breast cancer cells [18]. For that purpose, we silenced NF-κB/p65 using the siRNA-strategy and confirmed the knockdown at both transcript and protein levels, as previously reported [18]. Here, we questioned its role in other biological processes, focusing on understanding distinct molecular breast cancer subtypes.

Initially, we evaluated the endogenous expression of NF-κB/p65 in our models, which was detectable in all studied cell lines (Figure S2). Then, we conducted the same NF-κB-silencing protocol described in our previous article [18].

**Figure 1:**
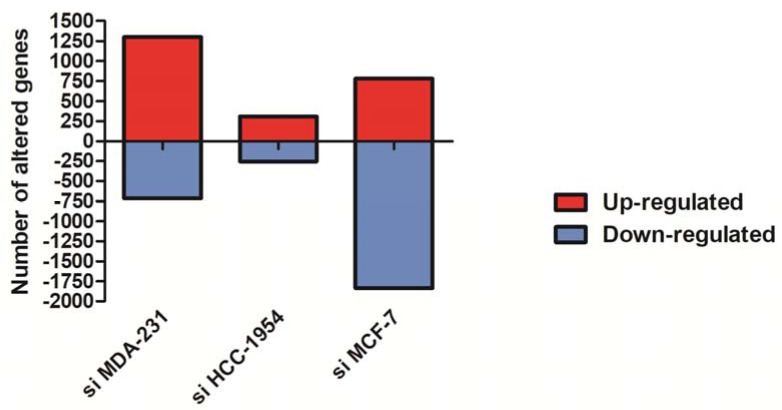
Differentially expressed genes identified by the chip array assay showing increased and decreased genes in breast cancer cells with silenced nuclear factor-kappa B (NF-κB)/p65 compared with their scramble counterparts. Positive values (red columns) correspond to the number of up-regulated genes, and negative values (blue columns) correspond to the number of down-regulated genes.

To determine the global gene expression profile in NF-κB-silenced human breast cancer cells, we performed a comparative transcriptome analysis by expression chip array assay. Using this approach, transcript levels of NF-κB knockdown in MDA-MB-231, HCC-1954 and MCF-7 cells were used and compared with their scramble counterparts from each cell line. As a consequence of NF-κB/p65 knockdown, considering a ≥1.5-fold change as the cut-off to define up- or down-regulation, 2017 genes were differentially expressed in NF-κB/p65-silenced (siNF-κB) MDA-MB-231, 564 genes in NF-κB/p65-silenced (siNF-κB) HCC-1954, and 2614 genes in NF-κB/p65-silenced (siNF-κB) MCF-7 cells. The total numbers of increased and decreased genes are shown in Figure 1. Notably, more down-regulated genes were found in MCF-7 cells, while the most up-regulated genes were found in MDA-MB-231 cells.

As each studied cell line corresponded to one specific BC subtype, different genes were altered in response to NF-κB silencing. Through a pathway enrichment analysis using MetaCore software v.6.35 (Thomson Reuters, Toronto, Canada), we found *Oxidative stress cellular signaling* among the Top 20 pathways most representative in all differentially expressed gene lists: siNFkB MDA-MB-231 (*p*-value = 1.180 × 10^−7^), siNFkB HCC-1954 (*p*-value = 4.294 × 10^−3^), and siNFkB MCF-7 (*p*-value = 4.557 × 10^−4^). We also identified pivotal members of the redox balance using the PANTHER and KEGG gene ontology tools, as described in Table 2. Thus, the focus of the validation dataset was on OS experiments to understand biological significance of OS for the intrinsic BC phenotypes during NF-κB silencing. 

**Figure 2:**
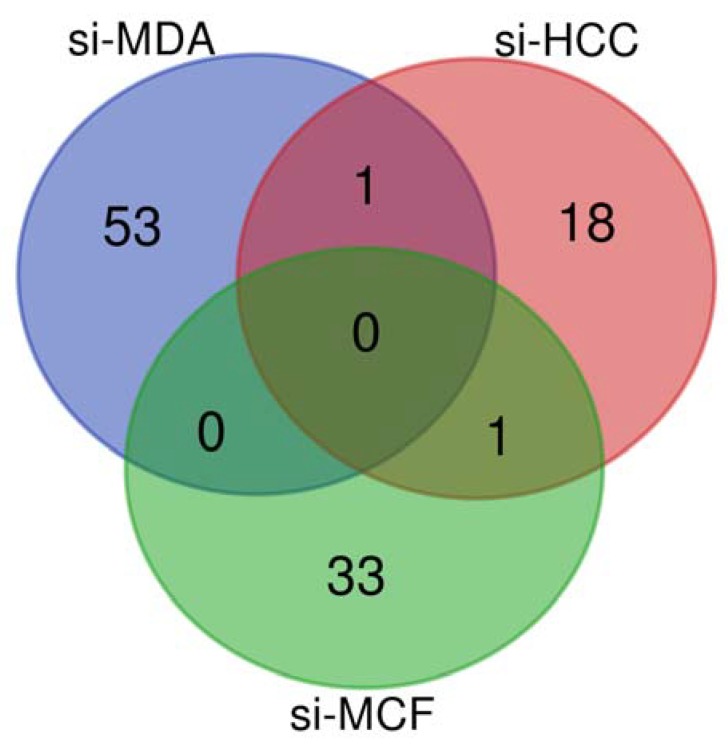
Venn diagram based on the lists of altered genes related to redox metabolism found in breast cancer models silenced for NF-κB/p65 compared with their scramble counterparts. Si-MDA: NF-κB/p65-silenced MDA-MB-231; si-HCC: NF-κB/p65-silenced HCC-1954; and si-MCF: NF-κB/p65-silenced MCF-7.

In silico analysis of these results showed that the genes that were altered in siNF-κB MDA-MB-231 were related to redox metabolism, such as aldo-keto reduction (*AKR1B10*, *AKR1C2*), glutathione metabolism (*GPX2*, *GPX3*, *GSTM1*, *GSTT1*, *GSTT2*, *GSTK1*, *GSTP1*), NADH dehydrogenase activity (*NDUFB9*, *NDUFS6*), the PI3K family (*PIK3C2B*, *PIP4K2C*, *PITPNC1*), prostaglandin signaling (*PTGER4*, *PTGES*, *PTGFRN*, *PTGS2*), and the cytochrome P450 family (*POR*, *CYP2S1*, *CYP2UI*, *CYP4B1*, *CYP4Z1*, *CYB5A*). In addition, the potent hydroxylase xanthine dehydrogenase (*XDH*) was up-regulated, revealing an antioxidant effect. The altered genes in siNF-κB HCC-1954 were mainly related to the COX family (*COX11P1*, *COX8C*) and fatty acid metabolism (*FAR1*, *LIPK*). Finally, we identified members of the PI3K family (*PIK3C2A*, *PIK3CA*) and phospholipid metabolism (*PLCD1*, *PLA2G1B*, *PLA2G15*, *PLDG*, *PLCB4*) as the main modified genes in siNF-κB MCF-7. In addition, important genes related to the response to OS were found among the altered genes in siNF-κB MCF-7, such as *OXR1* and *TMX1*. Although none of the altered genes were common to all lists of redox metabolism-related genes, *HPGD* was found in the siNF-κB HCC-1954 and siNF-κB MDA-MB-231 lists, while *PEX1* was found in the siNF-κB HCC-1954 and siNF-κB MCF-7 lists (as presented in the Venn diagram, Figure 2). Interestingly, DNA repair members related to the response to OS were identified in all three altered gene lists: up-regulation of *TP63* and *RAD51* in siNF-κB MDA-MB-231, up-regulation of *BRCA1*, *RB1* and *XRCC4* in siNF-κB HCC-1954, and down-regulation of *BRCA2*, *ATM* and *ATR* in siNF-κB MCF-7.

**Figure 3:**
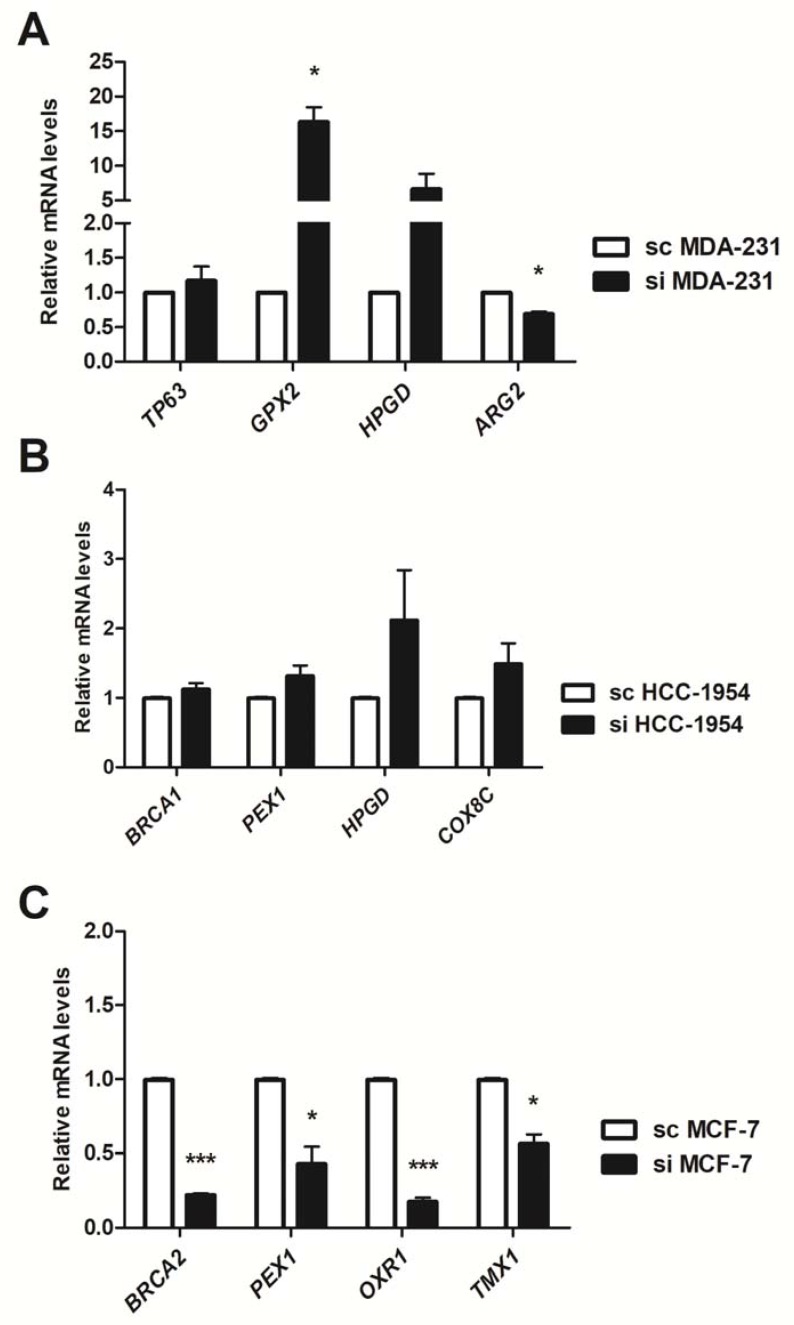
Relative expression by real time PCR (qPCR) of differentially expressed genes in the microarray analysis after NF-κB genetic silencing. The mRNA levels were assessed in MDA-MB-231 (**A**); HCC-1954 (**B**) and MCF-7 (**C**) cells, comparing the NF-κB-silenced condition (si) with the Scramble (sc) counterpart. Data are expressed as the means and standard errors of the means. *: *p*-value < 0.05; **: *p* < 0.01; ***: *p* < 0.001.

To confirm the microarray results, we selected some altered genes for mRNA level evaluation by RT-qPCR. As shown in Figure 3, we confirmed the microarray data: *TP63*, *GPX2*, *HPGD* were up-regulated and *ARG2* was down-regulated in siNF-κB MDA-MB-231 (Figure 3A); *BRCA1*, *PEX1*, *HPGD* and *COX8C* were up-regulated in siNF-κB HCC-1954 (Figure 3B); and *BRCA2*, *PEX1*, *OXR1* and *TMX1* were down-regulated in siNF-κB MCF-7 (Figure 3C).

### 3.2. Oxidative Stress Analyses

**Figure 4:**
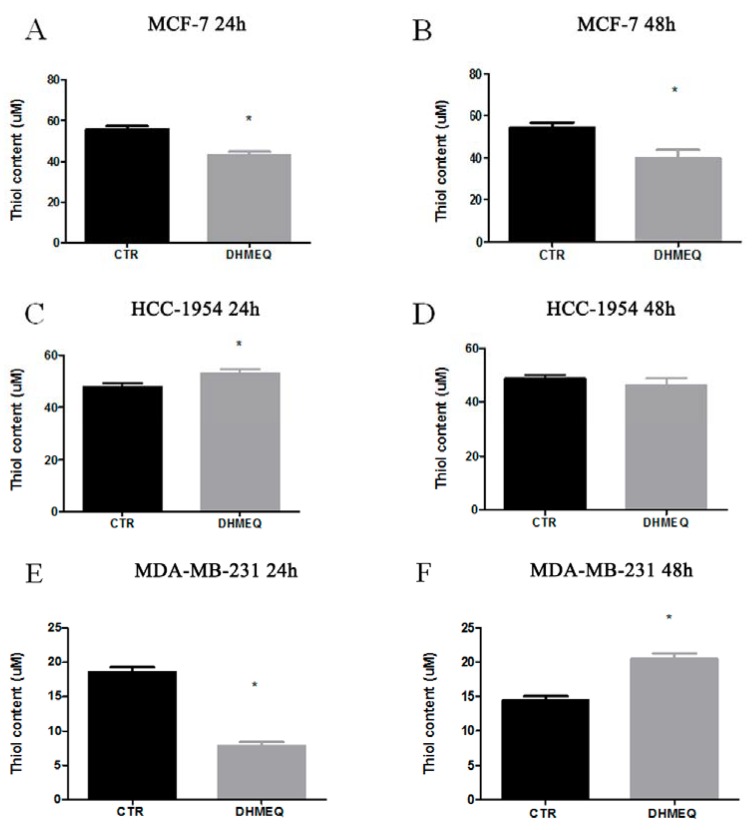
Thiol content. MCF-7 (**A**,**B**), HCC (**C**,**D**) and MDA-MB231 (**E**,**F**) cells treated (DHMEQ column) or not (CTR column) with NF-κB inhibitor for 24 or 48 h. Data are expressed as the means and standard errors of the means. * indicates statistical significance (*p* < 0.05).

To evaluate the relationship between NF-κB/p65 and OS in each BC subtype models, we treated the cells with the NF-κB inhibitor DHMEQ as described previously [18]. Initially, we evaluated the basal levels of lipid peroxidation, thiol, and NO in untreated cells (Figure S1). Regarding the thiol content, we observed that all BC cells presented distinct basal levels. DHMEQ treatment resulted in reduced thiol in MCF-7 cells after both durations of exposure (from 55.83 ± 1.5 µM to 43.05 ± 1.67 µM in 24 h, *p* = 0.0048, Figure 4A, and from 54.3 ± 2.5 µM to 39.72 ± 3.95 µM in 48 h, *p* = 0.0357, Figure 4B). For HCC-1954 cells, DHMEQ treatment led to an increased thiol content only after 24 h of exposure (from 47.7 ± 1.35 µM in controls to 53.16 ± 1.27 µM in treated cells, *p* = 0.0420, Figure 4C). For MDA-MB-231 cells, DHMEQ reduced the thiol content after 24 h (from 18.58 ± 0.72 µM in controls to 7.84 ± 0.55 µM in treated cells, *p* = 0.003, Figure 4E), while it augmented the thiol content after 48 h of exposure (14.4 ± 0.64 µM in controls and 20.45 ± 0.82 µM in the DHMEQ group, *p* = 0.0044, Figure 4F).

**Figure 5:**
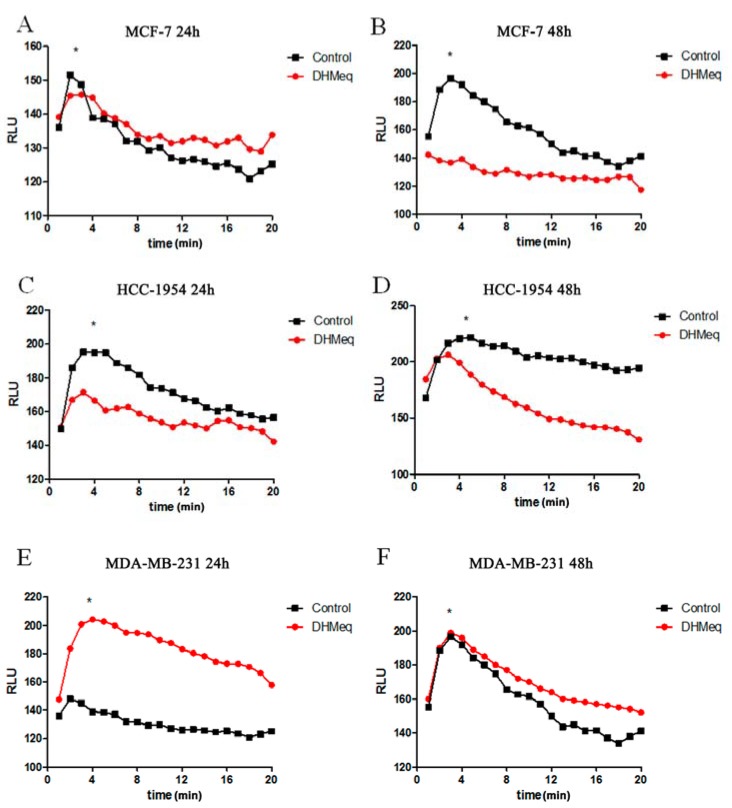
Lipid peroxidation profile. MCF-7 (**A**,**B**), HCC-1954 (**C**,**D**) and MDA-MB231 (**E**,**F**) cells treated or not with NF-κB inhibitor for 24 or 48 h. Data are expressed as the means. * indicates statistical significance (*p* < 0.05). Time (min).

Concerning the lipid peroxidation profile, it was noted that after 24 h of DHMEQ treatment, MCF-7 cells exhibited higher lipid peroxidation than the control (Figure 5A, *p* < 0.001), and this profile was reversed after 48 h (Figure 5B, *p* < 0.001). In HCC-1954 cells, DHMEQ treatment reduced lipid peroxidation after both periods of exposure (Figure 5C,D, *p* < 0.001). In contrast, MDA-MB231 cells presented enhanced lipid peroxidation after DHMEQ exposure after both durations of treatment (Figure 4E,F, *p* < 0.001).

**Figure 6:**
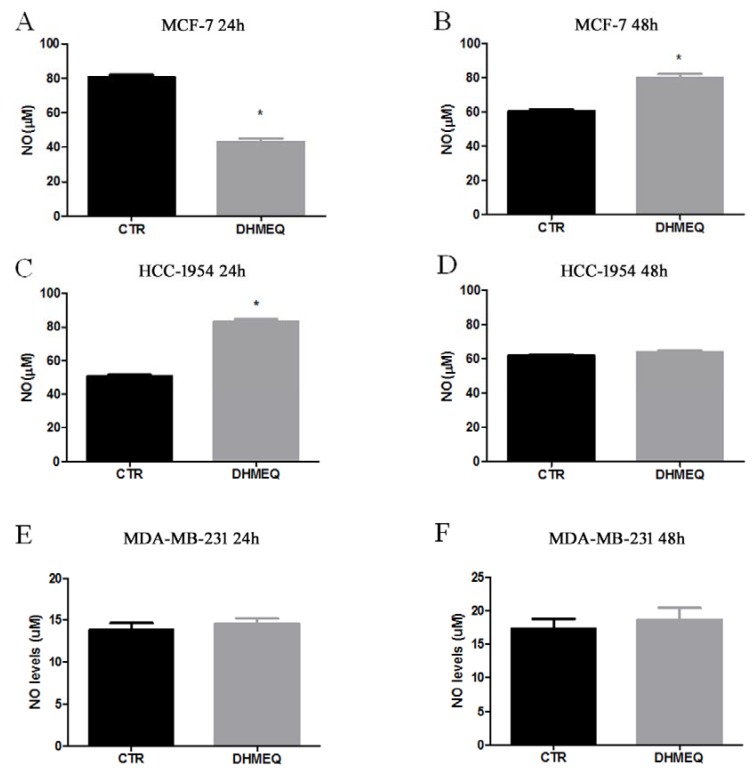
Nitrite as an estimate of NO levels. MCF-7 (**A**,**B**), HCC (**C**,**D**) and MDA-MB231 (**E**,**F**) cells treated (DHMEQ column) or not (CTR column) with NF-κB inhibitor for 24 or 48 h. Data are expressed as the means and standard errors of the means. * indicates statistical significance (*p* < 0.05).

The NO measurements indicated a reduction after 24 h of DHMEQ inhibition for MCF-7 (from 80.64 ± 1.43 µM in the control to 43.17 ± 1.82 µM in the DHMEQ group, *p* < 0.001, Figure 6A), followed by augmentation after 48 h of exposure (from 60.51 ± 1.06 µM in controls to 80 ± 2.18 µM in the DHMEQ group, *p* = 0.0013, Figure 6B). In HCC-1954 cells, only the 24-h treatment with DHMEQ altered NO (from 47.7 ± 1.35 µM in the control to 53.16 ± 1.26 µM in DHMEQ, *p* = 0.0420, Figure 6C). MDA-MB 231 cells did not show altered NO levels under any condition (Figure 6E,F).

## 4. Discussion

Oxidative stress (OS) is an indispensable condition to ensure genomic instability in cancer cells [26,27,28,29]. Thus, a redox imbalance represents an important factor for BC carcinogenesis and prognosis and is frequently associated with pro-inflammatory conditions [24,30,31,32]. Although a correlation between NF-κB and OS has been reported in cancer [33,34], its role in BC intrinsic groups has not been reported to date.

In the present study, we showed that NF-κB silencing altered the expression of a large number of genes in distinct subtypes of BC cells, some of which were shown to be important members of the redox balance through pathway enrichment analysis and gene ontology information. Relevant members involved in glutathione metabolism, prostaglandins, cytochrome P450 and cyclooxygenase were differentially expressed after NF-κB silencing in the distinct BC subtypes, suggesting a relationship between the redox balance and this transcription factor in such cells. 

When we analyzed individually each differentially expressed gene list, we observed that only peroxisomal biogenesis factor (*PEX*) 1 and 15-hydroxyprostaglandin dehydrogenase (*HPGD*) were differentially expressed in more than one list. *PEX1* was down-regulated in NF-κB-silenced MCF-7 and up-regulated in NF-κB-silenced HCC-1954. In these findings, because *PEX1* levels are in accordance with NF-κB levels, *PEX1* may play a pro-tumorigenic role in MCF-7 cells, which is supported by Bartling et al. [35] and Pang et al. [36], in opposition to what is observed in NF-κB-silenced HCC-1954 cells, as proposed by Lee et al. [37]. Different from *PEX1*, there was an accordance between *HPGD* up-regulation in NF-κB-silencing for MDA-MB-231 and HCC-1954, showing that *HPGD* may play a tumor suppressor role, as reported by Pham et al. [38] and Thiel et al. [39]. Additionally, high expression of *HPGD* is pointed out as favorable prognostic marker in renal cancer by The Human Protein Atlas [40].

Consequently, we validated the microarray dataset focusing on OS. To achieve this goal, we performed biochemical analyses assessing the antioxidant capacity, lipid peroxidation profile and NO status in BC cell subtypes and correlated such parameters with the presence or absence of NF-κB. Among the current small molecules able to inhibit NF-κB, DHMEQ is known for its effectiveness and specificity [41,42]. It directly binds to NF-κB/p65 and represses its nuclear translocation as well as DNA-binding activity [43], in addition to showing interesting effects against several tumors, including breast cancer [11,18,44]. Thus, the activity of this molecule as an NF-κB/p65 inhibitor was utilized in our study. 

First, we evaluated the basal levels of untreated cells. Lipid peroxidation was similar along time (24 h and 48 h). HCC-1954 cells were the most oxidized lineage when compared to both MCF-7 and MDA-MB-231, indicating that HER2 amplification may promote this event. Studies showed that cells overexpressing HER2 exhibit more lipid peroxidation than other subtype models [45], although patients carrying HER2-amplified tumors showed reduced lipid peroxidation in plasma. This contradiction has been discussed by other authors, and they suggested that the sustained oxidative stress may favor the change HER2-positive phenotype to a HER2-negative [46]. Both MCF-7 and MDA-MB-231 exhibited a very similar pattern of lipid peroxidation (Figure S1), although experimental and clinical studies have proposed that ER positive tumors are inducers of oxidative stress and lipid peroxidation [47,48,49], while others have reported that some oxidative stress-related markers are generally down-regulated in TNBC [50,51]. MDA-MB-231 also presented low basal levels of NO and thiol content when compared to the other cells, with enhanced depletion of antioxidant content after 48 h (Figure S1).

Regarding the NF-κB-inhibition effects, our data revealed completely distinct oxidation statuses of the three studied cell lines. MCF-7 cells, in which the main regulatory axis is ER signaling, displayed higher antioxidant capacity at basal levels when compared to the other cells, as observed by the attenuated lipid peroxidation profile and high thiol content. Such a profile may be related to the antioxidant activity of estrogen-induced signaling, which blocks reactive oxygen species (ROS) [52] and increases NO synthesis [53]. Furthermore, microarray data from MCF-7 cells revealed a few relevant members of the antioxidant response, indicating that ER signaling should explain the observed redox status. Despite the power of estrogen-induced signaling, our results indicated that the initial NF-κB inhibition observed after 24 h affected the redox status of MCF-7 cells, suggesting that the integrity of this axis might contribute to the redox stability of luminal cancer. Despite the mutual NF-κB/ER interaction in MCF-7 cells, ER signaling seemed to predominate in this case [54]. Sustained NF-κB inhibition seemed to potentiate the antioxidant capacity of MCF-7, since lipid peroxidation was even more attenuated after 48 h of treatment, in association with augmented NO. It has been demonstrated that NO can inhibit NF-κB binding to DNA by modifying the transcription factor structure [55], impairing its pro-oxidant capacity.

HER2-amplified cells demonstrated an intermediary oxidative profile when compared to the other BC cells investigated at basal levels, potentially because HER2 overexpression attenuates OS in breast cancer [32,49]. The 24-h NF-κB inhibition simultaneously augmented the thiol content and reduced lipid peroxidation, revealing a role for this transcription factor in the regulation of the OS of HER2-amplified cells. HER2 amplification is known to activate NF-κB in breast cancer through its canonical pathway [15], consequently triggering OS-related responses [56]. Thus, when we silenced NF-κB in these cells, this pro-oxidant arm was probably restricted, culminating in reduced OS. After 48 h, the attenuated oxidative profile was maintained, as noted by the reduced lipid peroxidation profile exhibited by DHMEQ-treated cells.

TNBC cells showed lower levels of antioxidants at basal levels, exhibiting a pro-oxidant status after 24 h of DHMEQ exposure that was completely reversed after 48 h of NF-κB inhibition. It is anticipated that TNBC cells would have a distinct response pattern compared with the other cells due to their different histological origin. Despite the lack of a known potent signaling pathway to deal properly with redox variations [50,51], NF-κB-silenced MDA-MB-231 exhibited important components of the antioxidant arm of the redox balance in the microarray analysis, such as glutathione peroxidase (GPX2), glutathione transferases (GSTM1, GSTK1, GSTP1, GSTT1 and GSTT2), ceruloplasmin (CP) and selenoprotein (SEPW1) (Table 2). Overexpression of ROS scavenger enzymes in TNBC has been associated with a good prognosis [57]. NF-κB inhibition for 48 h restored the thiol content and equilibrated the differences in the lipid peroxidation profile observed between the treated sample and its control, although the treated group retained elevated peroxidation. These findings suggest that after NF-κB inhibition, these cells may activate a late compensatory pathway that restores the antioxidant capacity. Thus, there may be some compensatory mechanism in cells that triggers increased thiol production likely to contain stress, which is in accordance with the findings of Kim et al. [57], who reported that increased expression of enzyme scavengers is associated with a better prognosis.

It is important to highlight that cancer cells are under continuous oxidative stress and, thus, present mechanisms to overcome and adapt to this situation. Since mild oxidative stress is a pivotal condition to ensure genomic instability, cells will utilize necessary compensatory mechanisms to ensure this condition. As we showed, different breast cancer cell types exhibit distinct patterns of redox-related genes. Furthermore, NF-κB may represent an additional mechanism related to oxidative stress maintenance in these cells, which operates in various forms to mediate other important predominant signaling components present in cancer cells.

## Figures and Tables

**Table 1:** Primer sequences of the investigated genes.

Primer	Sequence
*ACTB* forward	5′-TTCCTTCCTGGGCATGGAGTC-3′
*ACTB* reverse	5′-AGACAGCACTGTGTTGGCGTA-3′
*GAPDH* forward	5′-ATTCCACCCATGGCAAATTC-3′
*GAPDH* reverse	5′-GGCGTGGATGGGTCTTTCA-3′
*BRCA1* forward	5′-GACAGAGGACAATGGCTTCC-3′
*BRCA1* reverse	5′-AGCTCCTGGCACTGGTAGAG-3′
*BRCA2* forward	5′-GCCGTACACTGCTCAAATCA-3′
*BRCA2* reverse	5′-TTTGAAGTCATCTGGGCTGA-3′
*TP63* forward	5′-GAGGTTGGGCTGTTCATCAT-3′
*TP63* reverse	5′-GAGGAGAATTCGTGGAGCTG-3′
*PEX1* forward	5′-TGACTGCACTTGGTCACACA-3′
*PEX1* reverse	5′-CTGTCCAGGTCGAAACATTG-3′
*HPGD* forward	5′-CCATTTGTCCAGGCTTTGTT-3′
*HPGD* reverse	5′-AATCAATGGTGGGTCCAAAA-3′
*ARG2* forward	5′-GGCTGAGGTGGTTAGCAGAG-3′
*ARG2* reverse	5′-ACAAAGGTCTGGGCAGTGTC-3′
*GPX2* forward	5′-TTGCAACCAATTTGGACATC-3′
*GPX2* reverse	5′-TTTTTGGACAAGGGTGAAGG-3′
*COX8C* forward	5′-GGAAATGGCTGTTGGACTTG-3′
*COX8C* reverse	5′-ACTGCTTCAGGTTGCCTAGC-3′
*OXR1* forward	5′-TTGGTGCGTTAGCATCTGAG-3′
*OXR1* reverse	5′-CAAATTCTCCTCCTCCACCA-3′
*TMX1* forward	5′-GCAGATTGCCTTTGTCCTTC-3′
*TMX1* reverse	5′-TTCTTCATCCGCCTCTTGTT-3′

**Table:** 

siNFκB MDA-MB-231				
Fold Change	Gene Symbol	Description	GO Biological Process	GO Molecular Function
25.9	*ALDH1A3*	aldehyde dehydrogenase 1 family, member A3	retinoic acid biosynthetic process, metabolic process	aldehyde dehydrogenase (NAD) activity; oxidoreductase activity
21.19	*AKR1B10*	aldo-keto reductase family 1, member B10 (aldose reductase)	retinoid metabolic process; cellular aldehyde metabolic process; steroid metabolic process; oxidation-reduction process	aldo-keto reductase (NADP) activity; oxidoreductase activity
11.78	*TP63*	tumor protein p63	replicative cell aging	DNA binding transcription factor activity
10.72	*DHRS9*	dehydrogenase/reductase (SDR family) member 9	androgen metabolic process; progesterone metabolic process; retinol metabolic process	alcohol dehydrogenase (NAD) activity; oxidoreductase activity; oxidoreductase activity
9.4	*PTGES*	prostaglandin E synthase	prostaglandin metabolic process; cyclooxygenase pathway; response to lipopolysaccharide; fatty acid metabolic process	glutathione binding; isomerase activity
8.23	*DHRS3*	dehydrogenase/reductase (SDR family) member 3	retinol metabolic process; oxidation-reduction process; metabolic process	nucleotide binding; dehydrogenase activity; electron carrier activity; oxidoreductase activity
7.02	*RDH10*	retinol dehydrogenase 10 (all-trans)	retinoid metabolic process	oxidoreductase activity
5.01	*GPX2*	glutathione peroxidase 2 (gastrointestinal)	oxidation-reduction process; response to oxidative stress; cellular oxidant detoxification	electron carrier activity; peroxidase activity; oxidoreductase activity
5.01	*PTGS2*	prostaglandin-endoperoxide synthase 2 (prostaglandin G/H synthase and cyclooxygenase)		peroxidase activity; lipid binding; enzyme binding; heme binding; metal ion binding; oxidoreductase activity
4.57	*PTGFRN*	prostaglandin F2 receptor inhibitor	lipid particle organization	protein binding
3.5	*AKR1C2*	aldo-keto reductase family 1, member C2; aldo-keto reductase family 1 member C2-like	lipid metabolic process; prostaglandin metabolic process; steroid metabolic process	oxidoreductase activity; carboxylic acid binding
3.48	*STS*	steroid sulfatase (microsomal), isozyme S	steroid metabolic process	steryl-sulfatase activity; sulfuric ester hydrolase activity; metal ion binding; catalytic activity; hydrolase activity
3.42	*GSTT1*	glutathione S-transferase theta 1	glutathione metabolic process; xenobiotic metabolic process; oxidation-reduction process	glutathione transferase activity; glutathione peroxidase activity; protein binding
3.13	*MAOA*	monoamine oxidase A	xenobiotic metabolic process; oxidation-reduction process	primary amine oxidase activity; oxidoreductase activity
3.05	*CYP4B1*	cytochrome P450, family 4, subfamily B, polypeptide 1	cellular aromatic compound metabolic process; exogenous drug catabolic process; oxidation-reduction process	iron ion binding; oxidoreductase activity
2.92	*PIK3C2B*	phosphatidylinositol-4-phosphate 3-kinase, catalytic subunit type 2 beta	phospholipid metabolic process; protein phosphorylation; inflammatory response	lipid kinase activity; ATP binding
278	*CP*	ceruloplasmin (ferroxidase)	copper ion transport; oxidation-reduction process;	ferroxidase activity; copper ion binding; chaperone binding; oxidoreductase activity; metal ion binding
2.62	*SQRDL*	sulfide quinone reductase-like (yeast)	sulfur amino acid metabolic process; cellular nitrogen compound metabolic process; oxidation-reduction process	sulfide:quinone oxidoreductase activity; oxidoreductase activity
2.52	*CYB5A*	cytochrome b5 type A (microsomal)	L-ascorbic acid metabolic process; response to cadmium ion; oxidation-reduction process	aldo-keto reductase (NADP) activity; enzyme binding; heme binding; metal ion binding
2.39	*SEPW1*	selenoprotein W, 1	cell redox homeostasis	antioxidant activity; selenium binding
2.38	*IDO1*	indoleamine 2,3-dioxygenase 1	immune system process; chronic inflammatory response	electron carrier activity; heme binding; metal ion binding; oxidoreductase activity
2.36	*NDUFB9*	NADH dehydrogenase (ubiquinone) 1 beta subcomplex, 9, 22 kDa	mitochondrial electron transport, NADH to ubiquinone; respiratory electron transport chain; oxidation-reduction process	protein binding; NADH dehydrogenase activity
2.35	*SRD5A3*	steroid 5 alpha-reductase 3	androgen biosynthetic process; oxidation-reduction process; protein glycosylation; lipid metabolic process	oxidoreductase activity
2.21	*BLVRB*	biliverdin reductase B (flavin reductase (NADPH))	heme catabolic process; oxidation-reduction process	riboflavin reductase (NADPH) activity; oxidoreductase activity
2.18	*NDUFS6*	NADH dehydrogenase (ubiquinone) Fe-S protein 6, 13 kDa (NADH-coenzyme Q reductase)	mitochondrial electron transport, fatty acid metabolic process; oxidation-reduction process	NADH dehydrogenase activity; electron carrier activity
2.16	*PTGER4*	prostaglandin E receptor 4 (subtype EP4)	immune response; adenylate cyclase-modulating G-protein coupled receptor signaling pathway	prostaglandin E receptor activity; protein binding; signal transducer activity; prostaglandin receptor activity
2.13	*IDH1*	isocitrate dehydrogenase 1 (NADP+), soluble	glyoxylate cycle; tricarboxylic acid cycle; isocitrate metabolic process; 2-oxoglutarate metabolic process	magnesium ion binding; isocitrate dehydrogenase activity; oxidoreductase activity
2.13	*UQCRB*	ubiquinol-cytochrome c reductase binding protein	oxidative phosphorylation; mitochondrial electron transport, oxidation-reduction process	protein binding
2.06	*BCKDHA*	branched chain keto acid dehydrogenase E1, alpha polypeptide	branched-chain amino acid catabolic process; oxidation-reduction process;	alpha-ketoacid dehydrogenase activity; carboxy-lyase activity; metal ion binding; oxidoreductase activity
2.06	*HPGD*	hydroxyprostaglandin dehydrogenase 15-(NAD)	fatty acid metabolic process; prostaglandin metabolic process	NAD binding; oxidoreductase activity;
2.06	*PIP4K2C*	phosphatidylinositol-5-phosphate 4-kinase, type II, gamma	phosphatidylinositol phosphorylation	ATP binding; nucleotide binding; kinase activity; transferase activity
2.05	*GCLC*	glutamate-cysteine ligase, catalytic subunit	response to oxidative stress; glutamate metabolic process; glutathione metabolic process	coenzyme binding; magnesium ion binding; glutamate-cysteine ligase activity; ATP binding
2.05	*TSTD1*	thiosulfate sulfur transferase (rhodanese)-like domain containing 1; F11 receptor	sulfide oxidation	protein binding; transferase activity
2.04	*XDH*	xanthine dehydrogenase	xanthine catabolic process; oxidation-reduction process; regulation of reactive oxygen species	iron ion binding; electron carrier activity; oxidoreductase activity, metal ion binding
2.02	*DHODH*	dihydroorotate dehydrogenase (quinone)	nucleotide biosynthetic process; response to organic cyclic compound	dehydrogenase activity; oxidoreductase activity
2.01	*PITPNC1*	phosphatidylinositol transfer protein, cytoplasmic 1	phospholipid transport	protein binding; lipid binding; phosphatidylinositol transporter activity
1.99	*CYP2S1*	cytochrome P450, family 2, subfamily S, polypeptide 1	xenobiotic metabolic process; oxidation-reduction process	iron ion binding; oxidoreductase activity; steroid hydroxylase activity
1.98	*RAD51*	RAD51 recombinase	DNA repair; DNA recombinase assembly	DNA binding; recombinase activity
1.75	*GSTK1*	glutathione S-transferase kappa 1	glutathione metabolic process; xenobiotic metabolic process; oxidation-reduction process	transferase activity; peroxidase activity; receptor binding; oxidoreductase activity
1.72	*HEBP2*	heme binding protein 2	mitochondrial membrane potential; mitochondrial membrane permeability; response to reactive oxygen species	protein binding
1.64	*POR*	P450 (cytochrome) oxidoreductase	xenobiotic metabolic process; response to nutrient	enzyme binding; hydrolase activity; electron transfer activity
1.63	*CYP4Z1*	cytochrome P450, family 4, subfamily Z, polypeptide 1	oxidation-reduction process	iron ion binding; heme binding; aromatase activity; oxidoreductase activity; metal ion binding
1.52	*PEX11A*	peroxisomal biogenesis factor 11 alpha	peroxisome organization; lipid metabolic process	protein binding; protein homodimerization activity
1.51	*COX6C*	cytochrome c oxidase subunit VIc	mitochondrial electron transport; hydrogen ion transmembrane transport	oxidase activity
−1.52	*NOXA1*	NADPH oxidase activator 1	superoxide metabolic process; regulation of hydrogen peroxide metabolic process	NADPH oxidase activity; enzyme binding
−1.55	*NOS1AP*	nitric oxide synthase 1 (neuronal) adaptor protein	nitric oxide biosynthetic process; apoptotic process	nitric-oxide synthase binding
−1.6	*CYP2U1*	cytochrome P450, family 2, subfamily U, polypeptide 1	xenobiotic metabolic process; arachidonic acid metabolic process; oxidation-reduction process	iron ion binding; oxidoreductase activity
−1.65	*GPX3*	glutathione peroxidase 3 (plasma)	response to lipid hydroperoxide; oxidation-reduction process; response to oxidative stress	glutathione peroxidase activity; transcription factor binding; selenium binding; oxidoreductase activity
−1.67	*GSTP1*	glutathione S-transferase pi 1	response to reactive oxygen species; glutathione metabolic process; xenobiotic metabolic process	glutathione transferase activity; nitric oxide binding; transferase activity
−1.67	*MT1A*	metallothionein 1A	cellular response to cadmium ion; cellular response to zinc ion	protein binding; metal ion binding
−1.73	*GSTT2*	glutathione S-transferase theta 2; glutathione S-transferase theta 2B (gene/pseudogene)	glutathione metabolic process; xenobiotic metabolic process;	glutathione transferase activity; transferase activity; protein binding
−1.74	*MSRB3*	methionine sulfoxide reductase B3	response to oxidative stress; protein repair; oxidation-reduction process	zinc ion binding; oxidoreductase activity
−1.76	*ARG2*	arginase 2	urea cycle; arginine metabolic process; nitric oxide biosynthetic process	arginase activity; metal ion binding; hydrolase activity
−2.56	*GSTM1*	glutathione S-transferase mu 1	glutathione metabolic process; xenobiotic metabolic process	glutathione transferase activity; enzyme binding

**Table:** 

siNFκB HCC-1954				
Fold Change	Gene Symbol	Description	GO Biological Process	GO Molecular Function
1.78	*HSD17B2*	hydroxysteroid (17-beta) dehydrogenase 2	steroid biosynthetic process; oxidation-reduction process	estradiol 17-beta-dehydrogenase activity; testosterone dehydrogenase activity
1.7	*COX11P1*	COX11 cytochrome c oxidase assembly homolog (yeast) pseudogene 1	mitochondrial electron transport, hydrogen ion transmembrane transport	cytochrome-c oxidase activity
1.64	*BRCA1*	breast cancer 1, early onset	DNA repair, double-strand break repair via homologous recombination	DNA binding, ligase activity
1.58	*FAR1*	fatty acyl CoA reductase 1	glycerophospholipid biosynthetic process; oxidation-reduction process	fatty-acyl-CoA reductase (alcohol-forming) activity; oxidoreductase activity
1.58	*RB1*	retinoblastoma 1	cell cycle checkpoint, G1/S transition of mitotic cell cycle	DNA binding transcription factor activity, enzyme binding
1.57	*NUDT4*	nudix (nucleoside diphosphate linked moiety X)-type motif 4	cyclic nucleotide metabolic process, inositol phosphate metabolic process	hydrolase activity, endopolyphosphatase activity, protein binding
1.56	*HPGD*	hydroxyprostaglandin dehydrogenase 15-(NAD)	fatty acid metabolic process, prostaglandin metabolic process	NAD binding, prostaglandin E receptor activity, oxidoreductase activity
1.54	*PEX1*	peroxisomal biogenesis factor 1	peroxisome membrane biogenesis; cellular lipid metabolic process	protein binding; protein homodimerization activity
1.53	*DHRS7C*	dehydrogenase/reductase (SDR family) member 7C	oxidation-reduction process	retinol dehydrogenase activity; oxidoreductase activity
1.52	*XRCC4*	X-ray repair complementing defective repair in Chinese hamster cells 4	DNA repair, double-strand break repair	DNA binding; protein binding; ligase activity
1.51	*COX8C*	cytochrome c oxidase subunit VIIIC	mitochondrial electron transport, hydrogen ion transmembrane transport	cytochrome-c oxidase activity
1.51	*PTGDS*	prostaglandin D2 synthase 21 kDa (brain)	prostaglandin biosynthetic process, fatty acid metabolic process	transporter activity, retinoid binding, fatty acid binding
−1.51	*PDLIM3*	PDZ and LIM domain 3	actin filament organization	cytoskeletal protein binding; zinc ion binding
−1.52	*PDK2*	pyruvate dehydrogenase kinase, isozyme 2	glucose metabolic process, protein phosphorylation	protein serine/threonine kinase activity, pyruvate dehydrogenase kinase activity
−1.66	*TCAP*	titin-cap	cardiac muscle contraction, protein complex assembly	titin binding; ion channel binding
−1.72	*DHRS12*	dehydrogenase/reductase (SDR family) member 12	oxidation-reduction process	oxidoreductase activity
−1.75	*HSD17B4*	hydroxysteroid (17-beta) dehydrogenase 4	fatty acid metabolic process, fatty acid beta-oxidation	oxidoreductase activity
−1.76	*CYP4Z2P*	cytochrome P450, family 4, subfamily Z, polypeptide 2, pseudogene	oxidation-reduction process	iron ion binding; oxidoreductase activity, heme binding
−1.82	*LIPK*	lipase, family member K	lipid catabolic process; lipid metabolic process	hydrolase activity
−1.88	*MT-TM*	mitochondrially encoded tRNA methionine	tRNA Aminoacylation	catalytic activity

**Table:** 

siNFκB MCF-7				
Fold Change	Gene Symbol	Description	GO Biological Process	GO Molecular Function
1.84	*NDUFS7*	NADH dehydrogenase (ubiquinone) Fe-S protein 7, 20 kDa (NADH-coenzyme Q reductase)	mitochondrial electron transport; oxidation-reduction process	NADH dehydrogenase activity; oxidoreductase activity
1.75	*PTGS1*	prostaglandin-endoperoxide synthase 1 (prostaglandin G/H synthase and cyclooxygenase)	prostaglandin biosynthetic process; fatty acid metabolic process	peroxidase activity; lipid binding; heme binding; metal ion binding; dioxygenase activity; oxidoreductase activity
1.67	*PLCD1*	phospholipase C, delta 1	phospholipid metabolic process; inositol phosphate metabolic process	calcium ion binding; phosphoric diester hydrolase activity; phosphatidylserine binding
1.61	*CYB561A3*	cytochrome b561 family, member A3	oxidation-reduction process	protein binding; oxidoreductase activity; metal ion binding
1.6	*PLA2G1B*	phospholipase A2, group IB (pancreas)	activation of MAPK activity; fatty acid biosynthetic process	phospholipase activity; receptor binding; calcium ion binding; hydrolase activity; metal ion binding
1.59	*PDE3B*	phosphodiesterase 3B, cGMP-inhibited	cAMP catabolic process; glucose homeostasis	metal ion binding; phosphoric diester hydrolase activity
1.57	*FTHL17*	ferritin, heavy polypeptide-like 17	iron ion transport	ferric iron binding; metal ion binding
1.57	*LPO*	lactoperoxidase	response to oxidative stress; hydrogen peroxide catabolic process; oxidation-reduction process	heme binding; metal ion binding; peroxidase activity; oxidoreductase activity
1.57	*TXNP6*	thioredoxin pseudogene 6		response to oxidative stress
1.56	*PLA2G15*	phospholipase A2, group XV	phospholipid metabolic process	phospholipase activity; hydrolase activity
1.54	*PLD6*	phospholipase D family, member 6	phospholipid metabolic process; nucleic acid phosphodiester bond hydrolysis	endoribonuclease activity; metal ion binding; endonuclease activity; hydrolase activity
1.52	*MSRB1*	methionine sulfoxide reductase B1	response to oxidative stress; protein repair; oxidation-reduction process	zinc ion binding; oxidoreductase activity; metal ion binding
1.51	*PEX16*	peroxisomal biogenesis factor 16	protein targeting to peroxisome; peroxisome organization	protein binding
1.5	*PRDX5*	peroxiredoxin 5	response to oxidative stress; inflammatory response; apoptotic process	thioredoxin peroxidase activity; antioxidant activity; receptor binding; oxidoreductase activity
1.5	*TP53RK*	TP53 regulating kinase	tRNA modification; protein phosphorylation; p53 binding	protein serine/threonine kinase activity; ATP binding; hydrolase activity; nucleotide binding
−1.51	*CYB5R4*	cytochrome b5 reductase 4	superoxide metabolic process; glucose homeostasis; oxidation-reduction process; NADP metabolic process	oxidoreductase activity; heme binding; metal ion binding
−1.53	*PEX1*	peroxisomal biogenesis factor 1	protein targeting to peroxisome; peroxisome organization	protein binding; ATP binding; nucleotide binding
−1.54	*DECR1*	2,4-dienoyl CoA reductase 1, mitochondrial	fatty acid metabolic process; oxidation-reduction process	NADPH binding; oxidoreductase activity
−1.56	*TXNDC9*	thioredoxin domain containing 9	cell redox homeostasis; biological process	protein binding
−1.61	*SEPP1*	selenoprotein P, plasma, 1	selenium compound metabolic process; response to oxidative stress	selenium binding
−1.62	*OXR1*	oxidation resistance 1	response to oxidative stress; oxidation-reduction process	protein binding; oxidoreductase activity
−1.63	*TMX1*	thioredoxin-related transmembrane protein 1	protein folding; cell redox homeostasis; oxidation-reduction process	disulfide oxidoreductase activity
−1.64	*PLCB4*	phospholipase C, beta 4	lipid metabolic process	calcium ion binding; phosphoric diester hydrolase activity; phospholipase C activity
−1.81	*CYCS*	cytochrome c, somatic	mitochondrial electron transport; apoptotic process	iron ion binding; electron transfer activity; heme binding
−2.1	*PIK3CA*	phosphatidylinositol-4,5-bisphosphate 3-kinase, catalytic subunit alpha	glucose metabolic process; 1-phosphatidylinositol-3-kinase activity	transferase activity; protein serine/threonine kinase activity; ATP binding
−2.19	*MDM1*	Mdm1 nuclear protein homolog (mouse)	p53 binding protein; regulation of centriole replication	protein binding
−2.76	*ATM*	ATM serine/threonine kinase; nuclear protein, ataxia-telangiectasia locus	DNA repair; telomere maintenance	transferase activity; DNA binding; protein serine/threonine kinase activity
−2.81	*PIK3C2A*	phosphatidylinositol-4-phosphate 3-kinase, catalytic subunit type 2 alpha	phosphatidylinositol biosynthetic process	phosphotransferase activity; ATP binding
−2.85	*ATR*	ATR serine/threonine kinase	DNA repair; cell cycle; DNA damage checkpoint	transferase activity; DNA binding; protein serine/threonine kinase activity
−3.04	*NUDT12*	nudix (nucleoside diphosphate linked moiety X)-type motif 12	NADP catabolic process	metal ion binding; hydrolase activity
−3.18	*CYP4F30P*	cytochrome P450, family 4, subfamily F, polypeptide 30	oxidation-reduction process	oxidoreductase activity
−3.57	*PYROXD1*	pyridine nucleotide-disulfide oxidoreductase domain 1	oxidation-reduction process	protein binding; oxidoreductase activity
−3.82	*BRCA2*	breast cancer 2, early onset	double-strand break repair via homologous recombination; DNA synthesis involved in DNA repair	protease binding; histone acetyltransferase activity; protein binding; H3 histone acetyltransferase activity; H4 histone acetyltransferase activity; gamma-tubulin binding; DNA binding
−3.88	*ATRX*	alpha thalassemia/mental retardation syndrome X-linked	DNA repair; nucleosome assembly	chromatin binding; helicase activity; DNA binding; DNA helicase activity; helicase activity

## Supplementary Materials

The following are available online at http://www.mdpi.com/2073-4425/9/7/320/s1, Figure S1. Endogenous levels of lipid peroxidation (A), thiol content (B) and NO (C) in the untreated condition of MCF-7, HCC-1954 and MDA-MB-231 cells at 24 and 48 h. Figure S2. Relative mRNA expression of NF-B/p65 in MCF-7, HCC-1954 and MDA-MB-231 cells.
