# Supplementary material for: NF-kappaB Regulates Redox Status in Breast Cancer Subtypes
Source: Genes (Basel). 2018 Jun 26;9(7):320. doi: 10.3390/genes9070320 (PMC6070792; doi:10.3390/genes9070320)
Supplement: Supplementary file 1 [file genes-09-00320-s001.pdf]

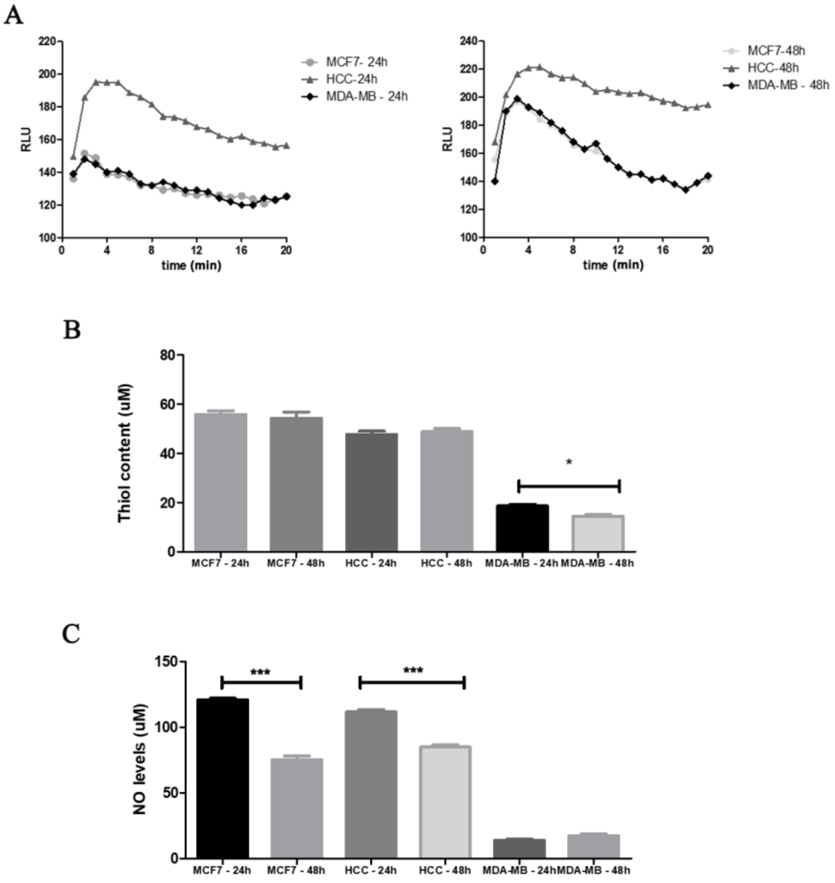

Figure S1. Endogenous levels of lipid peroxidation (A), thiol content (B) and NO (C) in the untreated condition of MCF-7, HCC-1954 and MDA-MB-231 cells at 24 and 48h. Data are expressed as the means and standard errors of the means. *: *p*-value < 0.05, **: *p* < 0.01, ***: *p* < 0.001.

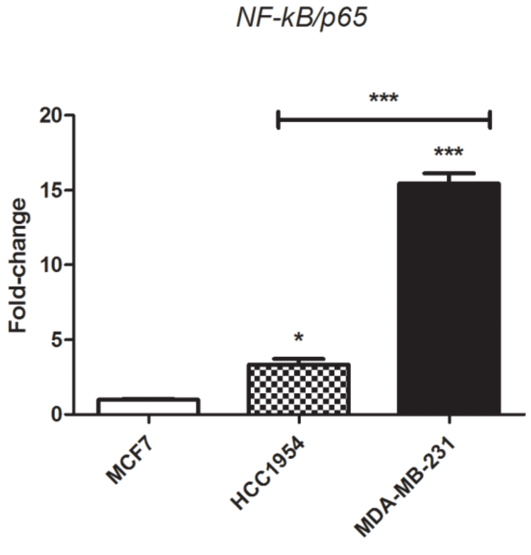

Figure S2. Relative mRNA expression of NF-B/p65 in MCF-7, HCC-1954 and MDA-MB-231 cells. The data were expressed as the mean ± SD. *: *p* < 0.05; ***: *p* < 0.001.
